# An extension of latent unknown clustering integrating multi-omics data (LUCID) incorporating incomplete omics data

**DOI:** 10.1093/bioadv/vbae123

**Published:** 2024-08-24

**Authors:** Yinqi Zhao, Qiran Jia, Jesse Goodrich, Burcu Darst, David V Conti

**Affiliations:** Department of Population and Public Health Sciences, Keck School of Medicine, University of Southern California, Los Angeles, CA 90033, United States; Department of Population and Public Health Sciences, Keck School of Medicine, University of Southern California, Los Angeles, CA 90033, United States; Department of Population and Public Health Sciences, Keck School of Medicine, University of Southern California, Los Angeles, CA 90033, United States; Public Health Sciences Division, Fred Hutch Cancer Center, Seattle, WA 98109, United States; Department of Population and Public Health Sciences, Keck School of Medicine, University of Southern California, Los Angeles, CA 90033, United States

## Abstract

**Motivation:**

Latent unknown clustering integrating multi-omics data is a novel statistical model designed for multi-omics data analysis. It integrates omics data with exposures and an outcome through a latent cluster, elucidating how exposures influence processes reflected in multi-omics measurements, ultimately affecting an outcome. A significant challenge in multi-omics analysis is the issue of list-wise missingness. To address this, we extend the model to incorporate list-wise missingness within an integrated imputation framework, which can also handle sporadic missingness when necessary.

**Results:**

Simulation studies demonstrate that our integrated imputation approach produces consistent and less biased estimates, closely reflecting true underlying values. We applied this model to data from the ISGlobal/ATHLETE “Exposome Data Challenge Event” to explore the association between maternal exposure to hexachlorobenzene and childhood body mass index by integrating incomplete proteomics data from 1301 children. The model successfully estimated proteomics profiles for two clusters representing higher and lower body mass index, characterizing the potential profiles linking prenatal hexachlorobenzene levels and childhood body mass index.

**Availability and implementation:**

The proposed methods have been implemented in the R package *LUCIDus*. The source code is available at https://github.com/USCbiostats/LUCIDus.

## 1 Introduction

Recent developments in biotechnologies have made omics data available for numerous cohort studies. For example, the Human Early-Life Exposome project (HELIX) measured molecular omics signatures, including DNA methylation, whole blood transcription, metabolites, and plasma proteins from 1301 children at the age of 6–11 in six European countries (Maitre *et al.* 2018). Such omics-rich cohort studies provide unprecedented opportunities to investigate the direct and indirect effects of exposures on complex disease phenotypes and to characterize the biological processes underlying these associations. Despite the potential, the non-independence and high dimensionality of multi-omics data bring up challenges in integrated statistical analysis, and innovative statistical methods are needed to address these issues.

Integrative genomic studies typically focus on linking the genome, epigenome, and transcriptome to a phenotype directly (Kristensen *et al.* 2014, Ritchie *et al.* 2015). Ritchie *et al.* summarized several existing methods and strategies of genomics integration including meta-dimensional and multi-staged analyses to enhance the understanding of the effects of genetics and genomics on complex outcomes. In contrast, environmental epidemiology studies with multi-omics data often aim to investigate patterns of multi-omics measurements, such as metabolites and proteins, and effects on a health outcome as a result of environmental exposures that precede current measurements or outcomes (Maitre *et al.* 2018, 2022, Jin *et al.* 2020, Stratakis *et al.* 2020, Wu *et al.* 2023). Guided by the underlying biology or the temporal sequence of measurements, these studies often share a common structure that relates the exposures to intermediate factors capturing transitional processes that ultimately result in an outcome. This suspected structure leads to analysis that can integrate multiple omics data acting on a disease or trait outcome *via* mediation or a latent structured model. Baccarelli *et al.* gave motivation for this type of precision environmental health in more detail (Baccarelli *et al.* 2023).

In terms of specific statistical methods for integrating multi-omics data, integrative clustering is a powerful and common approach to achieve dimension reduction while extracting key information (Pierre-Jean *et al.* 2020). An unsupervised clustering method called iCluster was proposed to conduct integrative clustering of multi-omics data using a joint latent variable model estimated by the expectation maximization (EM) algorithm (Shen *et al.* 2009, Shen *et al.* 2012, Mo *et al.* 2013). Pierre-Jean *et al.* also introduced other clustering methods including sparse generalized canonical correlation analysis (SGCCA) and similarity network fusion (SNF) (Tenenhaus *et al.* 2014, Wang *et al.* 2014). Besides clustering, other dimension reduction methods utilizing the decomposition of variance framework were proposed including joint and individual variation explained (JIVE), which functions as an extension of principal component analysis (PCA) (Lock *et al.* 2013). When taking the exposure into account, mediation models have been implemented to explore the underlying mechanism among exposures, multi-omics, and a phenotype. To link clustering approaches with environmental exposures often results in a two-step analysis in which clusters are estimated first and then subsequent mediation analysis is performed. Alternatively, high-dimensional mediation analysis may be directly performed. Song *et al.* extended their previous causal mediation analysis to high-dimensional multi-omics data by utilizing a Bayesian linear mixed model with continuous shrinkage priors on the key coefficients to obtain sparsity (Song *et al.* 2020a). Albert *et al.* and Derkach *et al.* presented useful statistical tools that incorporated building a latent variable model under the causal mediation framework (Albert *et al.* 2016, Derkach *et al.* 2019). Finally, to disentangle this complicated biological process, and effectively adopting the advantages of both clustering and mediation analysis, Peng *et al.* developed another model called latent unknown clustering integrating multi-omics data (LUCID). The LUCID model conducts integrative analysis linking omics data with exposomes and an outcome *via* a latent cluster to delineate distinct risk groups and exploit the underlying causal relationships among the variables of interest. This approach accounts for high-dimensional data by utilizing an $L_{1}$ penalty (Tibshirani 1996) to obtain a sparse solution and facilitate model interpretation (Peng *et al.* 2020). This model has successfully identified biologically relevant omics features which link exposures with different disease phenotypes (Jin *et al.* 2020, Kasper *et al.* 2020, Stratakis *et al.* 2020, Maitre *et al.* 2022, Matta *et al.* 2022, Wu *et al.* 2023). For integrative genomic studies in which environmental exposures are not the primary focus, the LUCID model remains valuable. For example, LUCID can be used with germline genetic variants such as single nucleotide polymorphisms (SNPs) or polygenic risk scores (PRS) as the exposures. In this context, LUCID distinguishes the effects of germline genetic variants as they precede other omics data, focusing on how the genetics influences multiple omics levels and ultimately impact the outcome. Moreover, LUCID model aids in statistical estimation, as this genetic component can be coded as binary or ordinal, while other omics features are often continuous and high-dimensional, which makes it inappropriate if naively integrated (Ritchie *et al.* 2015). Goodrich *et al.* provide a more detailed discussion of these various statistical approaches and their pros and cons in the context of multi-omic analysis (Goodrich *et al.* 2024).

A significant challenge in the integrative analysis of multi-omics data is the problem of missingness for the omics measurements. In large cohort studies with exposures and outcomes measured for all individuals, it is common for some omics data to not be available for all participants due to budget limits or other factors such as failure to extract samples (Little and Rubin 2019). In the HELIX study, e.g. urine/serum metabolomics data are available in 1198 children, while miRNA is available for only 941 children (Maitre *et al.* 2018). This scenario, in which omics measurements are only available in a subset of samples, is known as a list-wise missing pattern. List-wise missingness can arise either completely randomly or due to systematic factors related to other measured variables, such as exposomes and disease phenotypes. Therefore, list-wise missingness is considered a scenario of either missing completely at random (MCAR) or missing at random (MAR) (Little and Rubin 2019). Another commonly observed missing pattern is when missing values occur in omics measurements across both samples and features, potentially due to measurement error, insufficient sample availability, or experimental constraints (Song *et al.* 2020b). Such missing pattern is also assumed to be MCAR or at least MAR (Little and Rubin 2019), and is referred to as sporadic missingness. In practice, missingness in omics data across both samples and features is less likely to be MCAR or MAR but missing not at random (MNAR). For example, missing values might emerge because the actual levels are below or beyond the limit of detection (LOD) of the technology (Yu *et al.* 2014).

There are several statistical methods available for addressing non-list-wise missing omics data when they are MCAR and MAR. Imputation methods based on chained equations including predictive mean matching are implemented in the R package *mice* (Buuren and Groothuis-Oudshoorn 2011). Likelihood based methods, such as the EM algorithm, are also popular approaches given their ease of implementation and flexible statistical framework (Little and Rubin 2019) with several approaches implemented in the R package *missMethods* (Rockel 2022). For integrated analysis, the above approaches would have to be implemented in a two-stage process. For example, Scrucca *et al.* proposed a two-step approach by first imputing missing values under a general location model and then conducting a clustering algorithm on the imputed dataset within a Gaussian mixture model (GMM) (Schafer 1997, Scrucca *et al.* 2016). This is implemented in the R package *mclust*, Zhang *et al.* extended the EM algorithm under the framework of a GMM to conduct clustering and imputation of missing data simultaneously (Zhang *et al.* 2021). The above approaches for missing data are appropriate for sporadic missingness but for list-wise missing patterns, most of these methods will treat the missing rows as MCAR and randomly generate new observations based on their estimated correlation structures, which overlooks the underlying MAR mechanism that the missingness might be related to exposures and the outcome. As an alternative, complete-case analysis (i.e. limiting the analysis to only individuals with observations for all variables) is easy to implement but might not be viable if a large number of samples have missing data (Pigott 2001). In the context of the LUCID model, these methods are potentially less efficient as they do not effectively incorporate the information from the exposures and the outcome given the assumptions of LUCID that omics levels are associated with the exposures and the outcome.

In this article, we extend the previously proposed LUCID model for integrated omics analysis to address the problem of list-wise missingness (or missing rows) in omics data with the assumption of MCAR or at least MAR. We derive the joint likelihood for LUCID by allowing omics data to be potentially missing. We propose a likelihood partition method for list-wise missingness and an integrated imputation framework for sporadic missingness, with both approaches implemented within an EM algorithm for maximum likelihood estimation. Although implemented, sporadic missingness should be approached carefully as it also relies on the strong assumptions of MCAR or MAR. We evaluate the performance of our approach through extensive simulation studies and demonstrate the advantage of the proposed method over complete-case analysis and other imputation methods, particularly for list-wise missingness. Finally, to illustrate the practical usefulness of the proposed method for addressing list-wise missingness in real omics data, we evaluate the impact of prenatal hexachlorobenzene (HCB) on childhood body mass index (BMI) with the integration of proteomic measurements. This analysis uses the publicly available “challenge data” from the ISGlobal/ATHLETE “Exposome Data Challenge Event”, simulated from the HELIX data (Maitre *et al.* 2022).

## 2 Methods

### 2.1 LUCID with complete omics data

**Figure 1.**
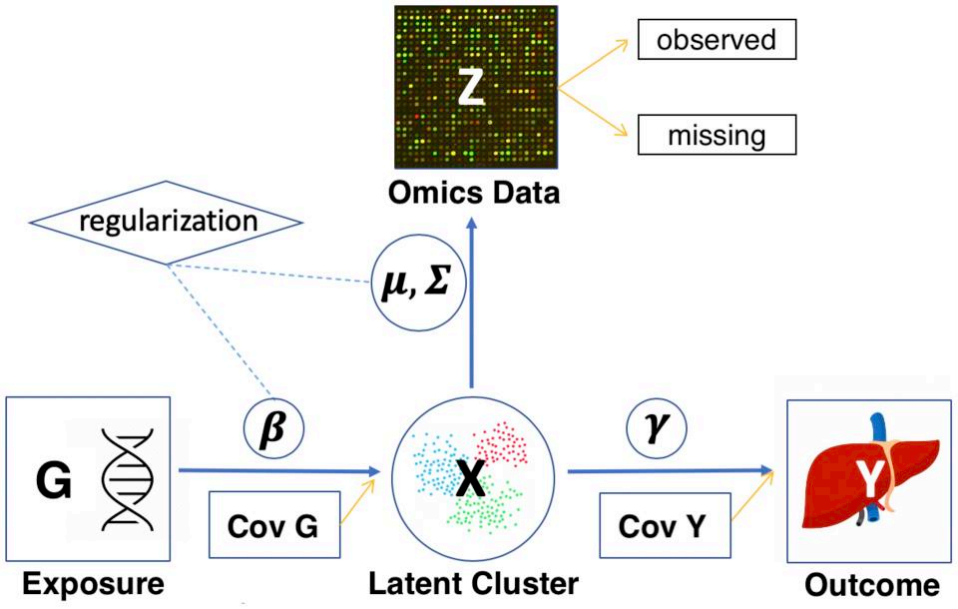
DAG of LUCID model. The squares represent observed data *G*, *Z*, and *Y*, the circles represent unobserved latent variables (clusters) and model parameters, and the diamond refers to $L_{1}\;$penalty terms for regularization. Cov *G* and Cov *Y* represent covariates to be adjusted in the LUCID model. Missingness is allowed in omics data and divided into subsets of observations with complete measurements and observations with missingness.

We first review the statistical framework of the LUCID model with complete omics data. LUCID jointly models the genomic/environmental exposures G, other omics data Z, and phenotype trait Y (Fig. 1). Suppose we have a sample of n observations indexed by i=1, 2, …, n. Let G be a n×p matrix with columns representing genetic or environmental exposures and rows being observations; Z be a n×m matrix of omics data with complete measurements; and Y be a n-length vector of phenotype trait.

The three data components (G, Z, and Y) are linked through a latent variable X consisting of k categories, each representing a latent cluster in the sample. In practice, k can arbitrarily set based on prior knowledge or *via* a grid search based on the overall model fit evaluated by the Bayesian information criterion (BIC).

The DAG in Fig. 1 implies the conditional independence among the distribution of X given G, Z given X, and Y given X. Additionally, we assume G, Z, and Y are measured through a prospective sampling procedure, so we do not model the distribution of G.

Since X is a discrete variable with k categories (indexed by j=1, 2, …, k), we assume that X follows a multinomial distribution conditioning on G, denoted by the softmax function S(X=j|G;β). We further assume that omics data Z follows a multivariate Gaussian distribution conditioning on X, denoted by $\phi (Z|X=j;\mu_{j},\Sigma_{j})$, where $\mu_{j}$ and $\Sigma_{j}$ are mean and variance–covariance matrices, respectively, for latent cluster j. This assumption fits in the model-based clustering framework (Fraley and Raftery 2002). To include more flexible geometric features of latent clusters, such as volume, shape, and orientation determined by $\Sigma_{j}$, we use the parameterization of variance–covariance matrices by the eigenvalue decomposition in the form of

(1) $${\;\Sigma }_{j}=\lambda_{j}D_{j}A_{j}D_{j}^{T}\;$$

where $\lambda_{j}$ is a scalar, $D_{j}$ is the orthogonal matrix of eigenvectors, and $A_{j}$ is a diagonal matrix whose values are proportional to eigenvalues (Banfield and Raftery 1993). The outcome Y is either a continuous or a binary variable. For illustration purposes, we assume Y is a continuous outcome following Gaussian distribution denoted by $\phi (Y|\gamma_{j},\;\sigma_{j}^{2})$ ($\gamma_{j}$ is cluster-specific effect and $\sigma_{j}^{2}$ is cluster-specific variance). The derivation for a binary outcome can be found elsewhere (Peng *et al.* 2020). We denote the observed data D={G, Z, Y}, the joint log-likelihood of the LUCID model is constructed as:

(2) $$l\left(\Theta |D\right)=\;\sum_{i=1}^{n}\log f\left(Z_{i},\;Y_{i}|G_{i};\Theta \right)=\sum_{i=1}^{n}\log\sum_{j=1}^{k}S\left(X_{i}=j|G_{i};\beta \right)\phi \left(Z_{i}\right|X_{i}=j;\mu_{j},\Sigma_{j})\phi (Y_{i}|\gamma_{j},\;\sigma_{j}^{2})\;$$

where Θ is the generic notation for all parameters in the LUCID model.

Because X is a latent variable, we use an EM algorithm to obtain the maximum likelihood estimator (MLE) of Θ in (2). We define $I(X_{i}=j)$ as an indicator function representing that observation i belongs to the latent cluster j. Then the log-likelihood function in (2) can be written as:

(3) $$\begin{array}{c} l\left(\Theta |D\right)=\;\sum_{i=1}^{n}\sum_{j=1}^{k}I\left(X_{i}=j\right)(\log S\left(X_{i}=j|G_{i};\beta_{j}\right)\\ \;+\log\phi \left(Z_{i}|\mu_{j},\;\Sigma_{j}\right)\;+\log\phi \left(Y_{i}|\gamma_{j},\;\sigma_{j}^{2}\right)) \end{array}$$

We define the responsibility, r, as the posterior inclusion probability (PIP) of observation i belonging to latent cluster j, given observed data and current estimations of Θ at iteration t, which is

(4) $$\begin{array}{c} r_{ij}^{\left(t\right)}=E\left(I\left(X_{i}=j\right)|D;\Theta^{\left(t\right)}\right)=P(X_{i}=j|G_{i},\;Z_{i},\;Y_{i};\Theta^{\left(t\right)})\\ \;=\frac{S\left(X_{i}=j|G_{i};\beta_{j}^{\left(t\right)}\right)\phi \left(Z_{i}|\mu_{j}^{\left(t\right)},\;\Sigma_{j}^{\left(t\right)}\right)\phi (Y_{i}|\gamma_{j}^{\left(t\right)},\sigma_{j}^{2(t)})}{\sum_{j^{\prime }=1}^{k}S\left(X_{i}=j^{\prime }|G_{i};\beta_{j^{\prime }}^{\left(t\right)}\right)\phi \left(Z_{i}|\mu_{j^{\prime }}^{\left(t\right)},\;\Sigma_{j^{\prime }}^{\left(t\right)}\right)\phi (Y_{i}|\gamma_{j^{\prime }}^{\left(t\right)},\sigma_{j^{\prime }}^{2(t)})} \end{array}$$

At iteration t, the *E*-step of the EM algorithm calculates the expectation of the complete data likelihood, denoted by $Q(\Theta |D,\;{\hat{\Theta }}^{\left(t\right)})$

(5) $$\;\begin{array}{c} Q\left(\Theta |D,\;{\hat{\Theta }}^{\left(t\right)}\right)=\;\sum_{i=1}^{n}\sum_{j=1}^{k}r_{ij}^{\left(t\right)}\log S\left(X_{i}=j|G_{i};\beta_{j}\right)\\ \begin{array}{c} \;+\sum_{i=1}^{n}\sum_{j=1}^{k}r_{ij}^{\left(t\right)}\log\phi \left(Z_{i}|\mu_{j},\;\Sigma_{j}\right)\\ \;+\sum_{i=1}^{n}\sum_{j=1}^{k}r_{ij}^{\left(t\right)}\log\phi \left(Y_{i}|\gamma_{j},\;\sigma_{j}^{2}\right) \end{array} \end{array}$$

The *M*-step maximizes (5) in terms of Θ, which results in the following estimations for iteration t+1:

(6) $$\beta_{j}^{\left(t+1\right)}=\arg\underset{\beta }{\max}\sum_{i=1}^{n}\sum_{j=1}^{k}r_{ij}^{\left(t\right)}\log S(X_{i}=j|G_{i};\beta_{j})$$

(7) $$\;\mu_{j}^{\left(t+1\right)}=\frac{\sum_{i=1}^{n}r_{ij}^{\left(t\right)}Z_{ij}}{\sum_{i=1}^{n}r_{ij}^{\left(t\right)}}\;$$

(8) $$\;\Sigma_{j}^{\left(t+1\right)}=\frac{\sum_{i=1}^{n}r_{ij}^{\left(t\right)}\left(Z_{ij}-\mu_{j}\right){\left(Z_{ij}-\mu_{j}\right)}^{T}}{\sum_{i=1}^{n}r_{ij}^{\left(t\right)}}\;$$

(9) $${\;\gamma }_{j}^{\left(t+1\right)}=\frac{\sum_{i=1}^{n}r_{ij}^{\left(t\right)}Y_{i}}{\sum_{i=1}^{n}r_{ij}^{\left(t\right)}}\;$$

(10) $$\;\sigma_{j}^{2(t+1)}=\frac{\sum_{i=1}^{n}r_{ij}^{\left(t\right)}{\left(Y_{i}-\gamma_{j}^{\left(t+1\right)}\right)}^{2}}{\sum_{i=1}^{n}r_{ij}^{\left(t\right)}}\;$$

Note that (8) is a closed-form solution for $\Sigma_{j}$ without any geometric constraints. Celeux and Govaert provide a detailed discussion of maximizing $\Sigma_{j}$, parameterized by the eigenvalue decomposition in (1) (Celeux and Govaert 1995). The R package *mclust* implements their algorithm (Scrucca *et al.* 2016), which we use to update $\Sigma_{j}$ in the *M*-step.

### 2.2 LUCID with missing omics data

#### 2.2.1 List-wise missing omics data

**Figure 2.**
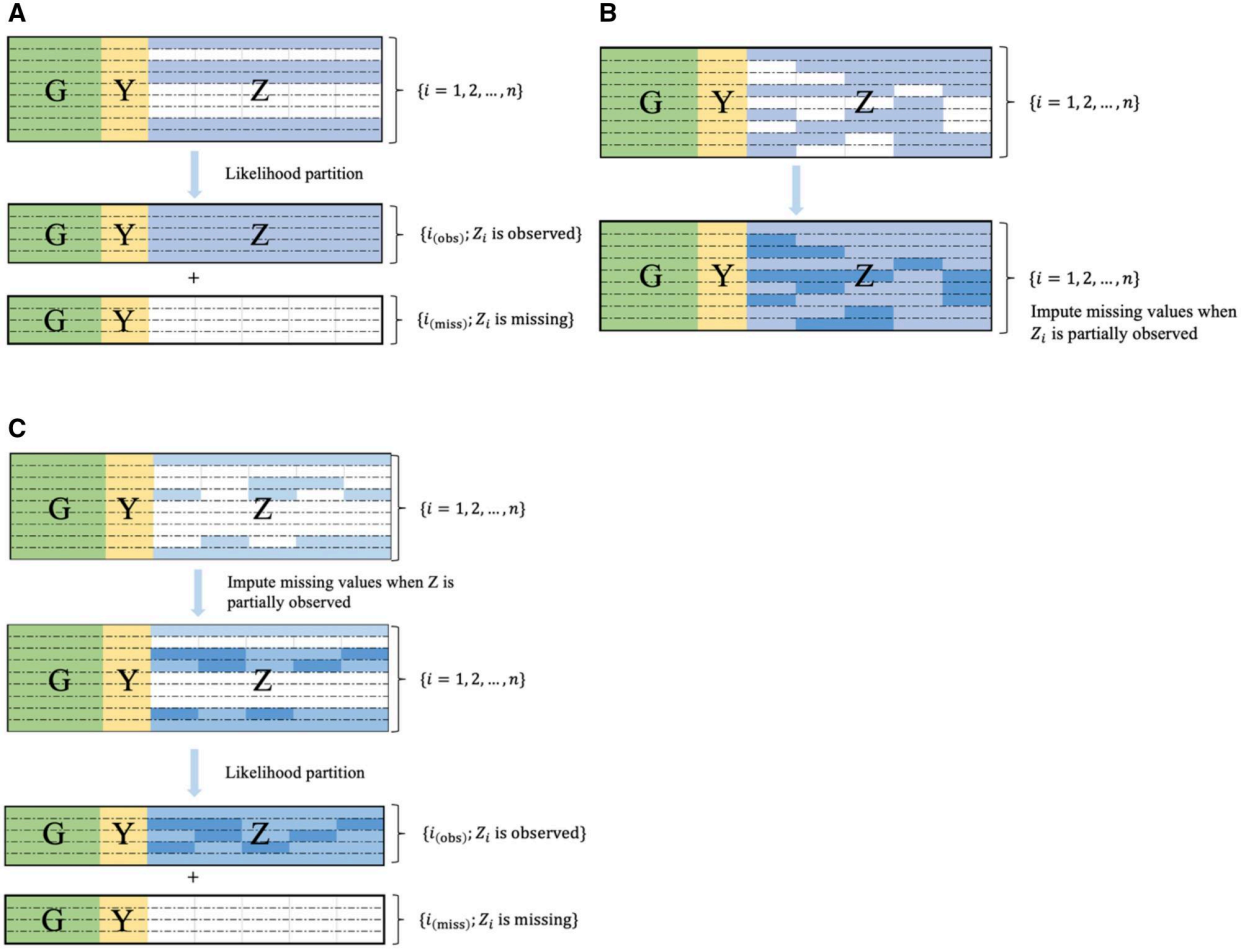
Missing patterns that LUCID assumes. (A) Illustration of the list-wise missing pattern; (B) illustration of the sporadic missing pattern; (C) illustration of a more general case with a combination list-wise and sporadic missing pattern.

To incorporate list-wise missing omics data in the LUCID model, we propose to use the likelihood partition technique illustrated in Fig. 2A. The observations are divided into two disjoint subsets: subset $\left\{i_{o}=1,\;2,\;\ldots ,\;n_{o}\right\}$ such that $Z_{i_{o}}$ is observed and subset $\left\{i_{m}=1,\;2,\;\ldots ,\;n_{m}\right\}$ such that $Z_{i_{m}}$ is completely missing. The likelihood function of the sample can be written as the sum of two components: (1) the joint likelihood of the subset $\left\{i_{o}\right\}$ denoted by $l_{o}(\Theta |D)$ and (2) the joint likelihood of the subset $\left\{i_{m}\right\}$ remains the same as (3), while that of the subset $\left\{i_{m}\right\}$ becomes

(11) $$\begin{array}{c} l_{m}\left(\Theta |D\right)=\;\sum_{i_{m}=1}^{n_{m}}\sum_{j=1}^{k}I\left(X_{i_{m}}=j\right)(\log S\left(X_{i_{m}}=j|G_{i_{m}};\beta_{j}\right)\\ +\log\phi \left(Y_{i_{m}}|\gamma_{j},\;\sigma_{j}^{2}\right)) \end{array}$$

We can obtain the MLE of Θ under a list-wise missing pattern *via* a modification of the E-step of the EM algorithm discussed in Section 2.1. Equation (11) explicitly points out that $l_{m}(\Theta |D)$ only consists of likelihood components related to G and Y. This results in the corresponding responsibility for the subset $\left\{i_{m}\right\}$, which is

(12) $$\begin{array}{c} r_{i_{m}j}^{\left(t\right)}=E\left(I\left(X_{i_{m})}=j\right)|{D;\Theta }^{\left(t\right)}\right)=P(X_{i_{m}}=j|G_{i_{m}},\;Z_{i_{m}},\;Y_{i_{m}};\Theta^{\left(t\right)})\\ \;=\frac{S\left(X_{i_{m}}=j|G_{i_{m}};\beta_{j}^{\left(t\right)}\right)\phi (Y_{i_{m}}|X_{i_{m}}=j;\gamma_{j}^{\left(t\right)},\sigma_{j}^{2(t)})}{\sum_{j^{\prime }=1}^{k}S\left(X_{i_{m}}=j^{\prime }|G_{i_{m}};\beta_{j^{\prime }}^{\left(t\right)}\right)\phi (Y_{i_{m}}|X_{i_{m}}=j^{\prime };\gamma_{j^{\prime }}^{\left(t\right)},\sigma_{j_{\prime }}^{2(t)})} \end{array}$$

For subset $\left\{i_{o}\right\}$, $r_{i_{o}j}^{\left(t\right)}$ is the same as (4). Therefore, in the *E*-step, the expectation of the log-likelihood of LUCID with list-wise missing omics data (denoted as Q(Θ|D)) can be partitioned into two parts

(13) $$\begin{array}{l} Q\left(\Theta |D\right)={El}_{o}\left(\Theta |D\right)+{El}_{m}\left(\Theta |D\right)\\ \;=\sum_{i_{o}=1}^{n_{o}}\sum_{j=1}^{k}r_{i_{o}j}^{\left(t\right)}(\log S\left(X_{i_{o}}=j|G_{i_{o}};\beta_{j}\right)+\log\phi \left(Z_{i_{o}}|X_{i_{o}}=j;\mu_{j},\Sigma_{j}\right)\\ +\log\phi (Y_{i_{o}}X_{i_{o}}=j;\gamma_{j},\;\sigma_{j}^{2}))\\ +\sum_{i_{m}=1}^{n_{m}}\sum_{j=1}^{k}r_{i_{m}j}^{\left(t\right)}\left(\log S\left(X_{i_{m}}=j|G_{i_{m}};\beta_{j}\right)+\log\phi \left(Y_{i_{m}}|X_{i_{m}}=j;\gamma_{j},\;\sigma_{j}^{2}\right)\right)\\ =\sum_{i=1}^{n}\sum_{j=1}^{k}r_{ij}^{\left(t\right)}\left(\log S\left(X_{i}=j|G_{i};\beta_{j}\right)+\log\phi \left(Y_{i}|X_{i}=j;\gamma_{j},\;\sigma_{j}^{2}\right)\right)\\ +\sum_{i_{o}=1}^{n_{o}}\sum_{j=1}^{k}r_{i_{o}j}^{\left(t\right)}\log\phi \left(Z_{i_{o}}|X_{i_{o}}=j;\mu_{j},\;\Sigma_{j}\right)\; \end{array}$$

In the *M*-step, the maximization of $\beta_{j}^{\left(t+1\right)}$, $\gamma_{j}^{\left(t+1\right)}$, and $\sigma_{j}^{2(t+1)}$ remains the same as (6), (9), and (10), respectively, since the likelihood components related to those parameters consist of all observations. We only need to replace $r_{ij}^{\left(t\right)}$ by $r_{i_{m}j}^{\left(t\right)}$ if $i\in \{i_{m}\}$. In contrast, the parameters associated with the omics data, $\mu_{j}$ and $\Sigma_{j}$, are updated only based on observations in subset $\left\{i_{o}\right\}$.

#### 2.2.2 Sporadic missing pattern

For sporadic missing omics data, the missing mechanism is ignorable with the assumptions of MCAR or MAR, and the EM algorithm is still applicable. To deal with the sporadic missing pattern in Z, we modify the two-step optimization algorithm for GMM with missing data proposed by Zhang *et al.* (2021) and integrate it into the EM algorithm for LUCID, as shown in Fig. 2B.

Suppose we have omics data $Z=\{Z_{1},\;Z_{2},\;\ldots ,Z_{i},\ldots ,\;Z_{n}\}$ with sporadic missing pattern, where $Z_{ia}$ represents observable variables for $Z_{i}$ and $Z_{ib}$ represents missing values. Under the LUCID model, we set the optimization problem as follows:

(14) $$\arg\underset{\Theta ,Z}{\max}l\left(\Theta |D=\{G,\;Z,\;Y\}\right)\;s.t.\;Z_{ia}\;\mathrm{is}\;\mathrm{fixed}\;\mathrm{for}\;\mathrm{all}\;i$$

We still use the EM algorithm discussed in Section 2.1 to optimize (14) iteratively. After initializing missing values in Z through imputation methods, the *E*-step and *M*-step remain the same at each iteration. After updating Θ, the problem is how to maximize the log-likelihood by imputing the missing part of Z given the observable part of Z fixed. According to (2), optimizing $l\left(\Theta |D\right)$ with respect to Z is only related to the likelihood component $\log\;\phi \left(Z|\mu ,\;\Sigma \right)$. Therefore, the optimization problem is equivalent to

(15) $$\arg\underset{Z}{\max}\sum_{i=1}^{n}\sum_{j=1}^{k}r_{ij}^{\left(t\right)}\log\phi \left(Z_{i}|{\;\mu }_{j}^{\left(t\right)},\;\Sigma_{j}^{\left(t\right)}\right)\;s.t.\;Z_{ia}\;\mathrm{is}\;\mathrm{fixed}\;\mathrm{for}\;\mathrm{all}\;i$$

Equation (15) can be divided into n sub-problems. Each sub-problem optimizes $Z_{i}$ with fixed $r_{ij}^{\left(t\right)},\;\mu_{j}^{\left(t\right)},\;\mathrm{and}\;\Sigma_{j}^{\left(t\right)}$. For each observation $Z_{i}$, we re-index it into observable and missing parts, $\left\{Z_{ia},\;Z_{ib}\right\}$. We divide cluster-specific mean $\mu_{j}$ and variance–covariance matrix $\Sigma_{j}$ the same way as $Z_{i}$, which is shown below:

(16) $$\mu_{j}=\left(\mu_{ja},\;{\;\mu }_{jb}\right),\;\Sigma_{j}^{-1}=\left(\begin{array}{cc} \Sigma_{\mathrm{jaa}}^{-1} & \Sigma_{\mathrm{jab}}^{-1}\\ \Sigma_{\mathrm{jba}}^{-1} & \Sigma_{\mathrm{jbb}}^{-1} \end{array}\right)$$

We then take the partial derivative of (15) in respective to $Z_{ib}$ and set it to 0. The closed-form solution is

(17) $$\begin{array}{c} Z_{ib}^{\left(t+1\right)}={\left(\sum_{j=1}^{k}r_{ij}^{\left(t\right)}p_{ij}^{\left(t\right)}\Sigma_{\mathrm{jbb}}^{-1\left(t\right)}\right)}^{-1}\cdot \;\\ \sum_{j=1}^{k}r_{ij}^{\left(t\right)}p_{ij}^{\left(t\right)}\left(\Sigma_{\mathrm{jba}}^{-1(t)}\mu_{ja}+\Sigma_{\mathrm{jbb}}^{-1(t)}\mu_{jb}-\Sigma_{\mathrm{jba}}^{-1(t)}Z_{ia}\right) \end{array}$$

where $p_{ij}^{\left(t\right)}=\phi (Z_{i}^{\left(t\right)}|\mu_{j}^{\left(t\right)},\Sigma_{j}^{\left(t\right)})$. Details of deriving (17) can be found in Zhang’s original paper.

Equation (17) implies that missing values in Z are updated at each iteration based on observed values and estimated parameters of GMM. Optimization of (15) is equivalent to a dynamic imputation process inside the LUCID framework. This modified EM algorithm obtains the MLE of Θ and imputes missing values simultaneously. As described by Zhang *et al.*, model parameters Θ and missing values in Z are updated by maximizing the expected likelihood function, which guarantees convergence of this modified EM algorithm.

#### 2.2.3 Combination of both missing patterns

We combine the methods in Sections 2.2.1 and 2.2.2 and extend LUCID to address both list-wise and sporadic missing patterns (Fig. 2C). If observation i has a sporadic missing pattern, we initialize missing values in $Z_{i}$ and treat $Z_{i}$ as “completely observable”. Next, we implement the likelihood partition to handle the remaining observations with a list-wise missing pattern. After calculating $\Theta^{\left(t\right)}$, we update the missing values $Z_{ib}$ using $\Theta^{\left(t\right)}$. We provide details of the EM algorithm to deal with the combination of list-wise and sporadic missingness in Algorithm 1. To initialize this modified EM algorithm, we use the R package *mix* to impute the missing values in *Z* under a general location model (Schafer 2022).

Algorithm 1. The EM algorithm for LUCID model with one latent variable and missing values in omics data **Z**

**Input**: Multi-view data **D**, total number of iterations $t_{\max}$, convergence tolerance $\epsilon_{\max}$,

1: **Initialization**:

2: Divide the index i into 3 subsets: (1) $i_{a}$: individuals with complete observation in **Z**; (2) $i_{b}$: individuals with partially observable values in **Z**; (3) $i_{c}$: individuals with complete missing values in **Z**.

3: Initialize $Z_{i_{b}}^{0}\;$by *mix*

4: Initialize $\mu^{0}$, $\Sigma^{0}$ by *mclust*

5: Initialize $\beta^{0}$ by *nnet*

6: Initialize$\;\gamma^{0}$ by *GLM*

7: Compute the log-likelihood *l_1_* by Equation (13)

8: ϵ ← ∞

9: t ← ∞ 

10: **EM algorithm:**

11: **while**$t\;<\;t_{\max}$**or**${\;\epsilon >\epsilon }_{\max}$**do**

12: **E-step**:

13: Compute $r_{i_{a}j}^{\left(t\right)}$, $r_{i_{b}j}^{\left(t\right)}$ based on Equation (4) and $r_{i_{c}j}^{\left(t\right)}$ based on Equation (12)

14: **M-step**:

15: Update $\beta^{\left(t+1\right)}$ by Equation (6)

16: Update $\mu^{\left(t+1\right)}$, $\Sigma^{\left(t+1\right)}\;$by *mclust*

17: Update $\gamma^{\left(t+1\right)}\;$by Equation (9)

18: **I-step**:

19: Update $Z_{i_{b}}^{\left(t+1\right)}\;$by Equation (17)

20: Compute the updated log-likelihood *l*_2_ using $\mu^{\left(t+1\right)}$, $\Sigma^{\left(t+1\right)}$, $\beta^{\left(t+1\right)}$, $\gamma^{\left(t+1\right)}$ according to Equation (13)

21: ϵ ← *l*_2_-*l*_1_

22: *l*_1_←*l*_2_

23: t ← t +1

24: **end while**

25: Compute $r_{ij}^{\left(t\right)}$ using $\mu^{\left(t\right)}$, $\Sigma^{\left(t\right)}$, $\beta^{\left(t\right)}$, $\gamma^{\left(t\right)}$

Output: $\Theta^{\left(t\right)}$ = {$\mu^{\left(t\right)}$, $\Sigma^{\left(t\right)}$, $\beta^{\left(t\right)}$, $\gamma^{\left(t\right)}$}, $r^{\left(t\right)}$ and $Z^{\left(t\right)}$

### 2.3 Software information

The described methods have been implemented in the R package *LUCIDus* which is available on CRAN (Zhao *et al.* 2022). The current version of *LUCIDus* is 3.0.1. *LUCIDus* can incorporate missing data, perform variable selection, obtain bootstrap confidence intervals, and visualize the LUCID model. It also includes a vignette covering the statistical background and example input data. Our implementation is based on the developer version of *LUCIDus*, which is available at https://github.com/USCbiostats/LUCIDus.

### 2.4 Simulation study

To showcase the robustness of the proposed integrated imputation method for handling list-wise missingness in omics data (Z), we performed comprehensive simulation studies across a range of missing ratios and compared the proposed method to other imputation methods in terms of their impact on the performance of the LUCID analysis. We generated 10 000 data points following the defined model in Fig. 1, conditional on pre-specified parameters and K=2 latent clusters characterizing low and high-risk groups. We selected K=2 for the ease of interpretation, but there may exist a more complex structure of the risk groups in the real-data analysis. Due to the conditional independence of the model, we first simulated 10 exposure variables (G). Conditional on G, we generated a cluster variable labeled X. Lastly, four omics variables Z and one outcome variable Y were generated conditional on X. For computational efficiency, we split the 10 000 observations into an 8000 sample training data set and a 2000 sample validation data set. Then, for every simulation iteration a random sample of 2000 observations was drawn from the 8000 sample data set and simulated list-wise missing pattern in Z over a grid of missing ratios. This data set was used as the training data and we analyzed the data using five methods: (1) the updated LUCID imputation framework (“L”); (2) the LUCID model based on a complete-case analysis (“complete-case”); (3) imputation using the location model implemented by the R package *mclust* (“impute-mclust”) followed by a LUCID analysis; (4) predictive meaning matching implemented by *mice* (“impute-mice”) followed by a LUCID analysis; and (5) EM imputation implemented by *missMethods* (“impute-EM”) followed by a LUCID analysis. For each resulting LUCID model, we compared parameter estimates to the simulated truth. Using the G and Z variables from the validation data set, we used the fitted LUCID model to predict cluster assignment and outcomes. We simulated 300 replications and examined several metrics to evaluate the performance of different methods, including mean parameter estimates and corresponding standard deviations compared to true simulated values and the accuracy of clustering using the area under the curves (AUC) by comparing estimated PIP to the known simulated cluster labels of the validation data set.

Though the proposed integrated imputation framework for sporadic missingness is regarded as an auxiliary functionality, we performed simulations studies under the same setting. Since it is infeasible to conduct complete-case analysis for sporadic missingness, the competing method (2) becomes the LUCID model based on the mean imputation (“impute-mean”). See Supplementary section A for results of simulation studies for sporadic missingness.

### 2.5 Applied data description and availability

We applied LUCID to the “challenge data” from the ISGlobal/ATHLETE “Exposome Data Challenge Event” held in April 2021. This dataset was created by a simulation based on the estimated correlation structure derived from the observed HELIX sub-cohort database. The data are available in the ExposomeDataChallenge2021 repository at https://github.com/isglobal-exposomeHub/ExposomeDataChallenge2021. The HELIX project is a multi-center longitudinal cohort study aimed at exploring the effects of early-life environmental exposures on health (Maitre *et al.* 2018). HELIX included 1301 mother–child pairs and measured 91 exposures in pregnancy and 116 exposures in childhood (Maitre *et al.* 2018). Children’s multi-omics profiles (methylome, transcriptome, proteins, and metabolites) were collected, but approximately 9% of the observations did not have complete data from four of the omics layers. Relying on imputing the missing omics data with the proposed method, we implemented LUCID to explore the underlying causal relationships between prenatal hexachlorobenzene (HCB) on childhood BMI with the integration of proteomic measurements. See Supplementary section B for more details on the applied analysis.

## 3 Results

### 3.1 Simulation study

**Figure 3.**
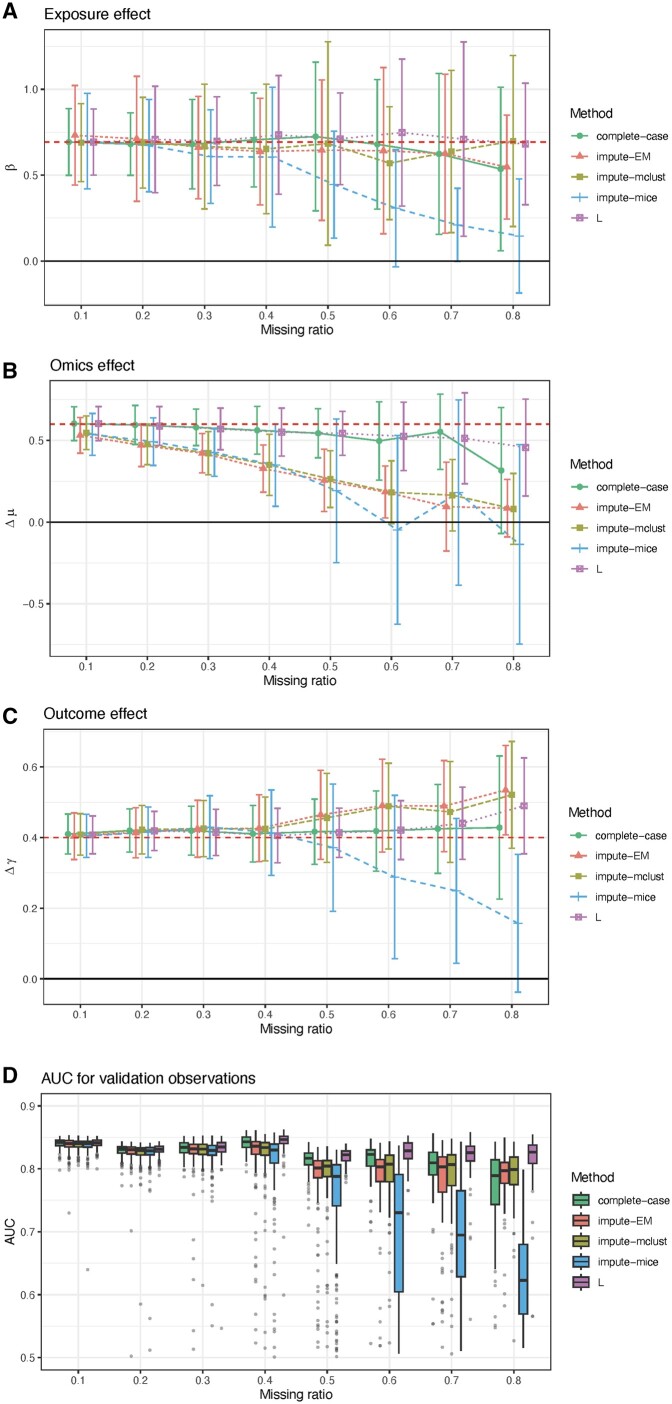
Simulations results for the list-wise missing pattern. (A) Exposure effect; (B) omics effect; (C) outcome effect; (D) AUC for validation observations. The horizontal dashed line on each plot represents the ground-truth effect.

Figure 3 shows the simulation results of the list-wise missingness across an increasing missingness ratio in the omics data Z from 0.1 to 0.8. For the exposure effect (the association between G and X), as the missing ratio in omics data increases, the average parameter estimates of *L* center around the true effect (indicated by the red dashed line) while the average parameter estimates of impute-mice are drastically biased towards 0, especially when the missing ratio is larger than 0.5. Impute-EM, impute-mclust, and impute-mice produce uniformly more biased estimates than *L* for high missing ratios. Regarding the uncertainty in estimation, standard deviations (SDs) show that uniformly all the methods behave similarly across scenarios (Fig. 3A). For the omics effect (the association between X and Z), *L* and complete-case consistently yield relatively unbiased estimates even when the missing ratio is high (>0.6), whereas impute-EM, impute-mclust, and impute-mice exhibit biased estimates even at a low missing ratio (>0.2). When the missing ratio is extremely high (0.8), a complete-case is more biased than *L*. All other methods yield comparable, and considerably smaller SDs than impute-mice. Notably, *L* demonstrates consistently smaller SDs, particularly when the missing ratio is less than 0.5 (Fig. 3B). Similar trends are observed when estimating the outcome effect (the association between X and Y). Both *L* and complete-case produce less biased estimates of the outcome effect than other methods, and the SDs of *L* are smallest across most of the missing ratios (Fig. 3C). *L* results in an obvious improved model performance in discriminating clusters compared to other methods in the validation set without using the outcome information. When the missing ratio increases, the median AUCs for *L* remain the most stable and the highest, whereas the median AUCs of complete-case, impute-EM, and impute-mclust drop moderately and the median AUCs of impute-mice drop drastically. The SDs of *L* is consistently the smallest, particularly when the missing ratio is high (Fig. 3D). In general, impute-EM, impute-mclust, and impute-mice present more biased estimations, relying solely on using the estimated correlation structures of observed rows to impute unobserved rows. While complete-case offers satisfactory estimates under the assumption of MCAR as missing rows are not dependent on G and Y, it remains inferior to *L*, particularly in scenarios of high missing ratios. This discrepancy can be attributed to *L*’s effective utilization of information from both G and Y.

### 3.2 Human Early-Life Exposome

**Figure 4.**
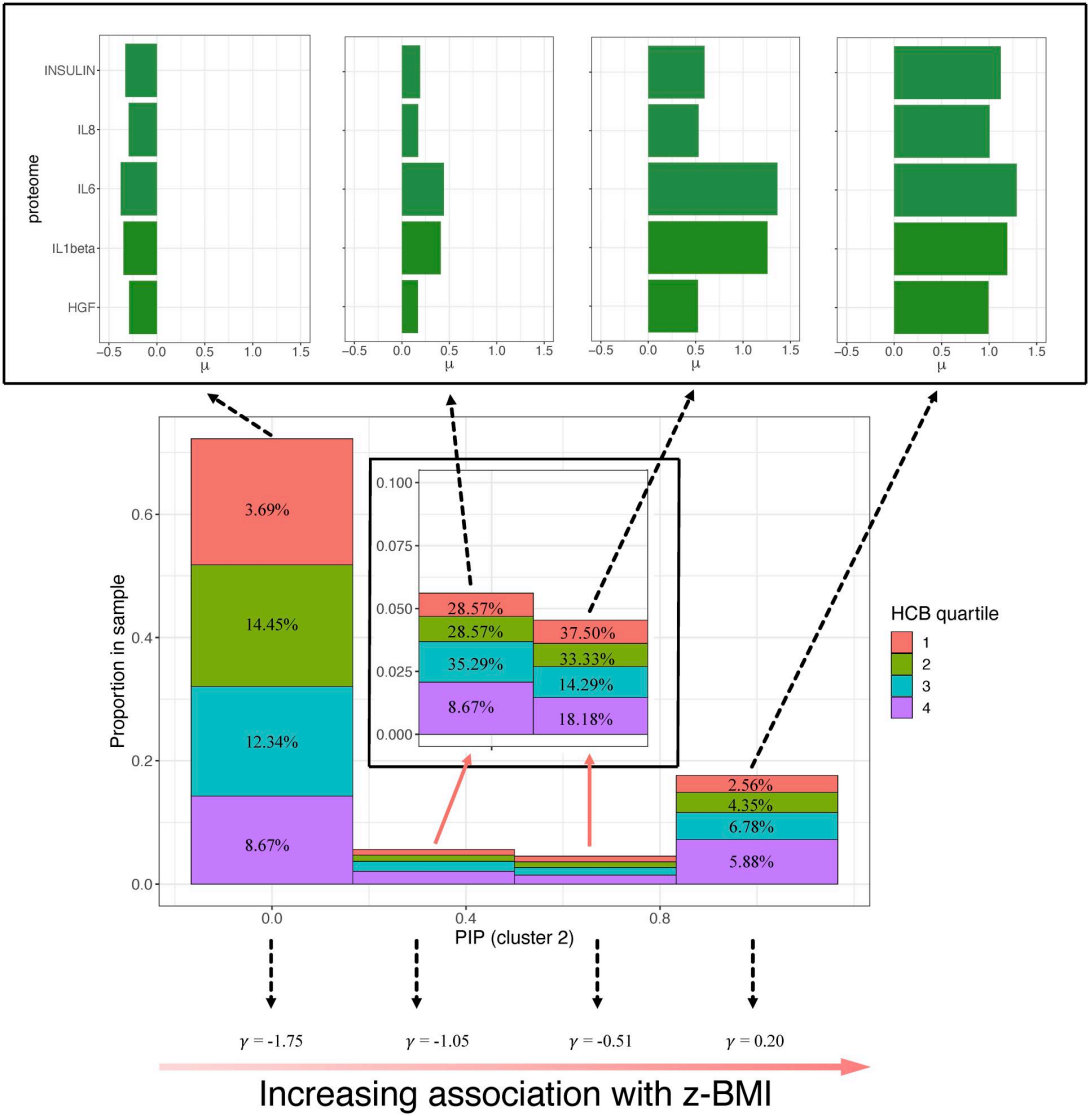
“Risk” profiles for individuals with and without measured proteomic data. The four bars from left to right on the histogram, each partitioned by the different HCB quartiles, indicate an increasing PIP to cluster 2, and they are also positively correlated with BMI *z*-score. The missingness ratio for each quartile on each bar is denoted. For each bar, the omics profiles (bar-specific) mean levels of proteomics are also presented.

**Figure 5.**
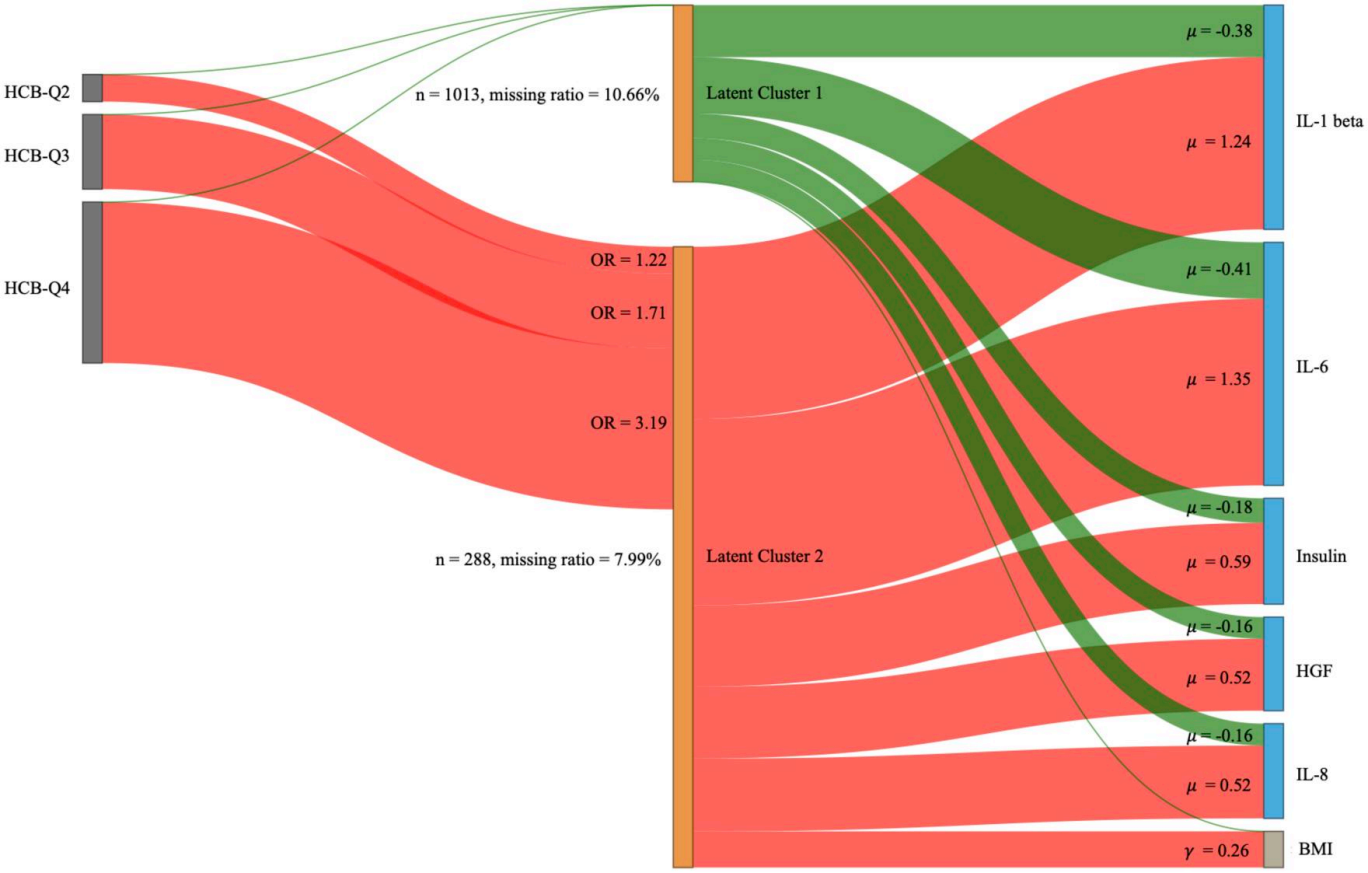
The Sankey diagram of the LUCID model fitted on the whole dataset with missingness. The nodes on the left represent the exposures of HCB quartiles, the middle nodes represent the latent clusters, and the nodes on the right represent the outcome of BMI z-score and proteomics. The width of the links and nodes corresponds to the effect size.

For the analysis of the entire dataset, including those with list-wise missing protein data, the supervised LUCID estimates two latent clusters after a grid search for the optimal number of clusters (Model 1). Latent cluster 2 was associated with a higher mean scaled BMI *z*-score ($\mu_{\mathrm{BMI},\;\mathrm{Cluster}\;1}=\;$-1.78, $\mu_{\mathrm{BMI},\;\mathrm{Cluster}\;2}=\;$-1.52). Table 1 presents the coefficient estimates for Model 1. Figure 4 presents a histogram visualizing the PIPs for cluster 2. The four bars in the histogram represent an increasing PIP corresponding to cluster 2, which also corresponds to an increasing association with BMI *z*-score. Each bar is partitioned by the HCB quartiles based on their proportions, and the missingness ratio for each quartile is denoted. The missingness ratios range from 2.56% to 37.50%. In addition, “risk profiles” (signatures of proteomic data) are constructed for observations within each bar by taking a weighted average of risk profiles for latent clusters 1 and 2, with weights determined by PIPs estimated from the LUCID analysis. Approximately 18.91% of the observations have PIPs greater or equal to 0.75 and are characterized by high expression levels of proteins. Most people (∼74.17%) fall into the first bar with PIPs less than 0.25, characterized by low expression levels of proteins. The two middle bars (∼6.92%) include individuals characterized by medium expression levels of proteins. Overall, proteomic expression levels increase with the PIPs for cluster 2. The associations between each bar and the *z*-BMI are also denoted, with an increasing association with the outcome as the PIP increases. Overall, a pattern emerges for the HCB quartiles, with lower quartiles having a higher proportion in the bins representing lower BMI/lower PIP, while higher quartiles have a higher proportion representing higher BMI/higher PIP. This trend is also reflected in Table 1 and Fig. 5, the Sankey diagram of the LUCID model fitted on the entire dataset, where an increasing HCB quartile is associated with latent cluster 2 (OR_HCB-second quartile_ = 1.2, OR_HCB-third quartile_ = 1.71 and OR_HCB-forth quartile_ = 3.19), which is ultimately associated with higher BMI. We also did a complete-case analysis to compare with our results, see Supplementary section C for details.

**Table 1.** The detailed coefficient estimates for Model 1.

	Cluster 1	Cluster 2
*N* (total = 1301)	1013	288
Missing ratio	10.66%	7.99%
HCB (exposure), odds ratio		
Quartile 1	–	Reference level
Quartile 2	–	1.22
Quartile 3	–	1.71
Quartile 4	–	3.19
Proteomics (omics data), scaled mean		
IL-1 beta	−0.38	1.24
IL-6	−0.41	1.35
IL-8	−0.16	0.52
Insulin	−0.18	0.59
HGF	−0.16	0.52
*Z*-score of BMI (outcome), mean	−1.78	−1.52

## 4 Discussion

In this article, under the assumptions of missingness patterns following MCAR and MAR, we develop an approach to handle list-wise missing values in an integrated omics analysis as an extension to the previous LUCID model. We also include an integrated imputation approach for MCAR or at least MAR sporadic missing values as an auxiliary feature. Using an integrated imputation process implemented within an EM algorithm, our proposed method handles list-wise missingness using a likelihood partition method and sporadic missingness by imputing the expected value at each iteration. Simulations showcase the potential advantages of the integrated imputation method for list-wise missingness in omics data in terms of the performance in coefficient estimation and clustering in the LUCID analyses as compared to traditional methods. In the real-data analysis, the integrated imputation method successfully identifies the list-wise missing pattern in the data and handles the missing values accordingly.

One underlying assumption of the LUCID model is that the missing omics data are MCAR or at least MAR for list-wise missingness and sporadic missingness, which implies that the missingness should be systemically related to observed variables such as other omics features, exposures, and the outcome, and cannot be related to unobserved variables. In practice, it is likely for list-wise missingness to be attributed to MCAR or MAR in large cohort omics studies, but it is less common for sporadic missingness to be MCAR or MAR. Sporadic missing values resulting from LOD or other MNAR scenarios remain a potential issue, and one way to mitigate this would be pre-imputing *via* existing methods appropriate for LOD missingness before analysis. A future potential direction for LUCID is to incorporate the detection limit mechanism for missing values *via* a truncated normal distribution to model the distribution of omics data.

An additional issue in the application of LUCID is the selection of the number of clusters, k, for the analysis. We have chosen to use BIC as it tends to select more parsimonious LUCID models by considering the increase in the number of parameters that occurs in other components of the LUCID model when the number of latent clusters increases. However, BIC has intrinsic limitations such as sensitivity to prior assumptions, dependence on sample size, and lack of flexibility. Additionally, alternative approaches for choosing the optimal k, such as the Elbow method, Silhouette analysis, and Gap statistic can be used in conjunction with the BIC to help ensure appropriate selection of the number of clusters (Rousseeuw 1987, Thorndike 1953, Tibshirani *et al.* 2001). However, these traditional approaches are also limited in the context of the LUCID model because they focus on how well the clusters explain the variabilities in the omics data Z given k, ignoring the other components of the LUCID model, and the issue that the number of corresponding parameters in these components changes as *k* changes. Overall, it is indispensable to conduct multiple analyses while considering the prior biological knowledge and the major research question to determine k, as there is no single best and unbiased approach regarding the LUCID model.

Overall, based on the results from simulations and a real-data analysis, the key advantages of an integrated analysis with the ability to impute missing data are evident. Often, different missing patterns (e.g. list-wise versus sporadic) need different imputation approaches commonly implemented by different software. However, the new implementation of the integrated method in the R package *LUCIDus* automatically identifies different missing patterns and conducts imputation and LUCID data analysis accordingly (Zhao *et al.* 2022). This extension enables the original LUCID model to be more versatile, convenient, and accurate when it comes to real-world data applications.

## Supplementary Material

vbae123_Supplementary_Data
