# Supplementary material for: An extension of latent unknown clustering integrating multi-omics data (LUCID) incorporating incomplete omics data
Source: Bioinform Adv. 2024 Aug 24;4(1):vbae123. doi: 10.1093/bioadv/vbae123 (PMC11368387; doi:10.1093/bioadv/vbae123)
Supplement: vbae123_Supplementary_Data [file vbae123_supplementary_data.zip › Supplement.docx]

**Supplement for “An Extension of Latent Unknown Clustering Integrating Multi-omics Data (LUCID) Incorporating Incomplete Omics Data” by Yinqi Zhao, Qiran Jia, Jesse Goodrich, Burcu Darst, and David V. Conti**

**Supplement A: Results of Simulation Studies for Sporadic Missingness**

The simulation results for sporadic missingness are presented in **Supplement Figure 1**. For the exposure effect (**Supplement Figure 1A**), with an increased missing ratio from 0.05 to 0.3, the average estimates across replicates for all methods remain stable, albeit slightly biased. Notably, impute-mean consistently demonstrates the most biased estimates, whereas L consistently exhibits the most unbiased estimates, particularly evident at high missing ratios (> 0.2). The SDs of all methods remain similar across various missing ratios while the SDs of impute-mean when the missing ratio is high (0.3) are large. A similar pattern is observed for the outcome effect (**Supplement Figure 1C**). The results of the omics effect are summarized in **Supplement Figure 1B**. While the mean estimation for the omics effect of L may deviate from the simulation truth as the missingness ratio in the omics data increases, it aligns closely with other methods. However, estimates from L exhibit slightly less bias when the missing ratio surpasses 0.25. Conversely, the mean estimation of impute-mean deviates from the true effect more dramatically. The SDs across all methods are consistent, while impute-mean demonstrates noticeably larger standard deviations particularly when the missing ratio is high (0.3). In terms of the AUC for the estimated clusters in the validation set, since all methods perform similarly well, maintaining consistently high AUC values and extremely low standard deviations across all missing ratios. For instance, L achieves a mean AUC of 0.94 with an SD of 0.0018 when the missing ratio is 0.2, with other methods demonstrating similar results. Overall, impute-mean produces more biased and uncertain estimations, while L shows slightly better performance for higher missing ratios compared to other methods.

**Supplement B: Details of Applied Data Analysis**

Even though it is banned in many countries, Hexachlorobenzene (HCB) is still a ubiquitous synthetic industrial chemical known to cause a wide range of adverse health effects, and prenatal exposure is known to induce changes in both infants and children (Cai et al., 2023; Reed et al., 2007). A previous study performed a random-effects meta-analysis and found that prenatal HCB levels were significantly associated with higher body mass index (BMI) z-scores in childhood (Stratakis et al., 2022). In our analysis, the goal is to conduct an integrative analysis using proteins to leverage the underlying causal relationships between prenatal HCB level and BMI in childhood. We included all the 1301 mother-child pairs with complete prenatal HCB level and BMI measurements in childhood. 131 of these 1,301 pairs had list-wise missing protein measurements. The continuous exposure variable, prenatal HCB level, was transformed into three indicator variables representing the second, third, and fourth quartiles, with the first quartile used as the reference. The outcome variable was the BMI z-score in childhood. We included five proteins of interest based on prior knowledge, namely Interleukin-1 beta (IL1beta), Interleukin-6 (IL6), Interleukin-8 (IL8), Insulin, and Hepatocyte Growth Factor (HGF). To account for potential confounding factors, five covariates were adjusted in the LUCID analysis for the association between latent clusters and the outcome: maternal pre-pregnancy BMI, maternal age, maternal education, child sex, and HELIX cohort. We implemented one supervised LUCID analysis on the entire data set and one supervised LUCID analysis on a subset of 1170 observations with complete omics data. For the entire data set, LUCID automatically imputed the missing omics data. For the subset, LUCID with complete omics data was implemented, which makes it a complete-case analysis. See **Supplement Figure 2** for the comparison of results between the proposed imputation method and the complete-case analysis. The resulting model is visualized via a Sankey diagram using the R package *networkD3* and a PIP figure with omics profiles.

**Supplement C: Comparison of Results between Complete-cases Analysis**

For comparison, we fitted a supervised LUCID model on the complete data only (Model 2). Supervised LUCID also identifies two latent clusters from a grid search, with latent cluster 2 being associated with higher mean scaled BMI z-score ($\mu_{BMI, Cluster 1}=$-1.73, $\mu_{BMI, Cluster 2}=$-1.51). **Supplement Figure 2** is a scatter plot comparing PIPs (cluster 2) of non-missing observations from Model 1 vs. PIPs (cluster 2) of all observations from Model 2 to examine whether the imputation of the missing omics data affects the PIPs for non-missing observations compared to complete-cases analysis. Although there exists more variation for PIP~0.5, the points correspond closely and indicate that the imputation of the missing omics data does not alter the PIPs for non-missing observations.

**Supplement Figure 1. Simulation results for the sporadic missing pattern.** A. Exposure effect; B. Omics effect; C. Outcome effect. The red dashed line on each plot represents the ground-truth effect.

**Supplement Figure 2:** the scatter plot of PIPs (cluster 2) of non-missing observations from Model 1 vs. PIPs (cluster 2) of all observations from Model 2.

References

Cai, A., Portengen, L., Govarts, E., Martin, L. R., Schoeters, G., Legler, J., . . . Remy, S. (2023). Prenatal exposure to persistent organic pollutants and changes in infant growth and childhood growth trajectories. *Chemosphere*, *314*, 137695. <https://doi.org/10.1016/j.chemosphere.2022.137695>

Reed, L., Buchner, V., & Tchounwou, P. B. (2007). Environmental toxicology and health effects associated with hexachlorobenzene exposure. *Rev Environ Health*, *22*(3), 213-243.

Stratakis, N., Rock, S., La Merrill, M. A., Saez, M., Robinson, O., Fecht, D., . . . Chatzi, V. L. (2022). Prenatal exposure to persistent organic pollutants and childhood obesity: A systematic review and meta-analysis of human studies. *Obes Rev*, *23 Suppl 1*(Suppl 1), e13383. <https://doi.org/10.1111/obr.13383>
