# Supplementary figures and images for: An extension of latent unknown clustering integrating multi-omics data (LUCID) incorporating incomplete omics data
Source: Bioinform Adv. 2024 Aug 24;4(1):vbae123. doi: 10.1093/bioadv/vbae123 (PMC11368387; doi:10.1093/bioadv/vbae123)

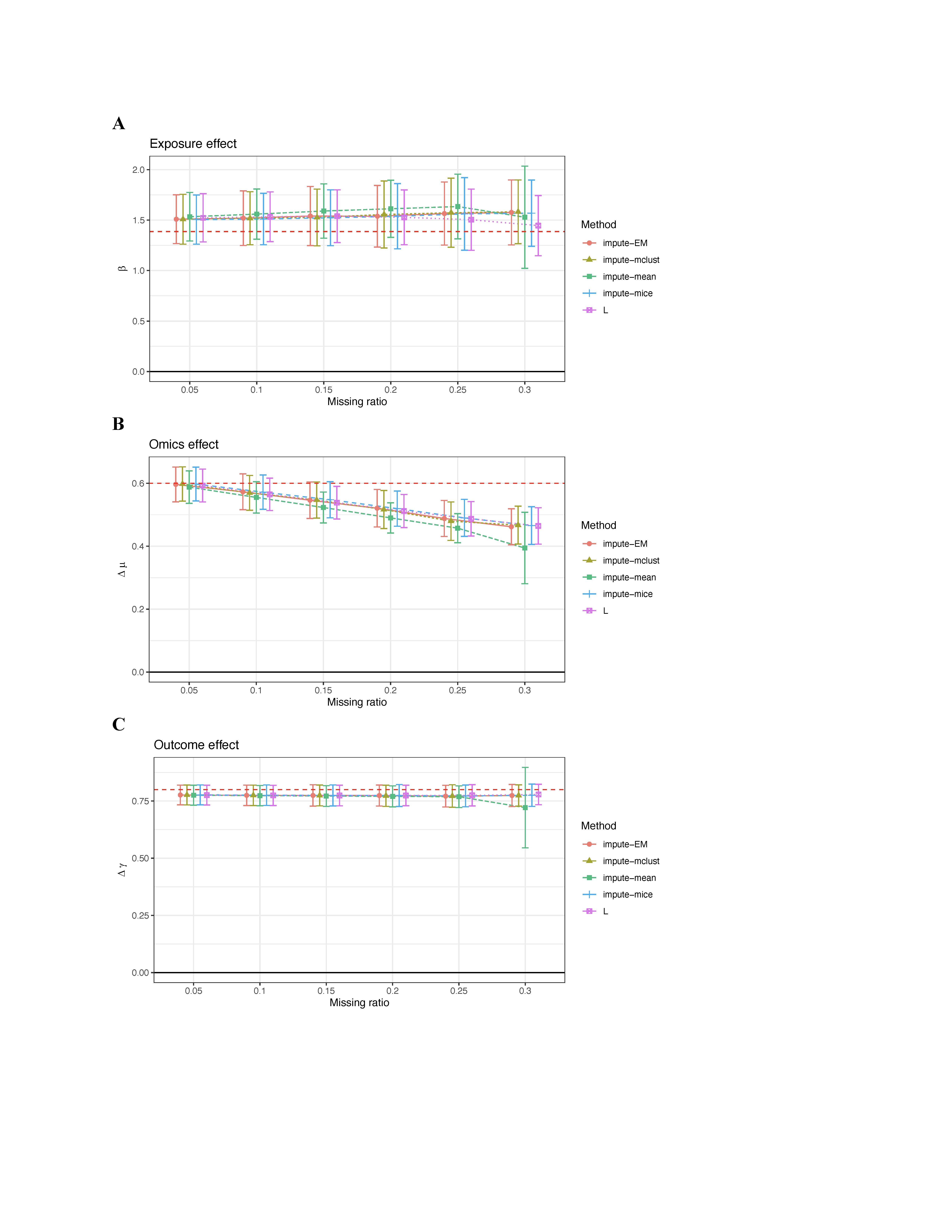

Supplement: vbae123_Supplementary_Data [file vbae123_supplementary_data.zip › Supplement Figure 1.tiff]

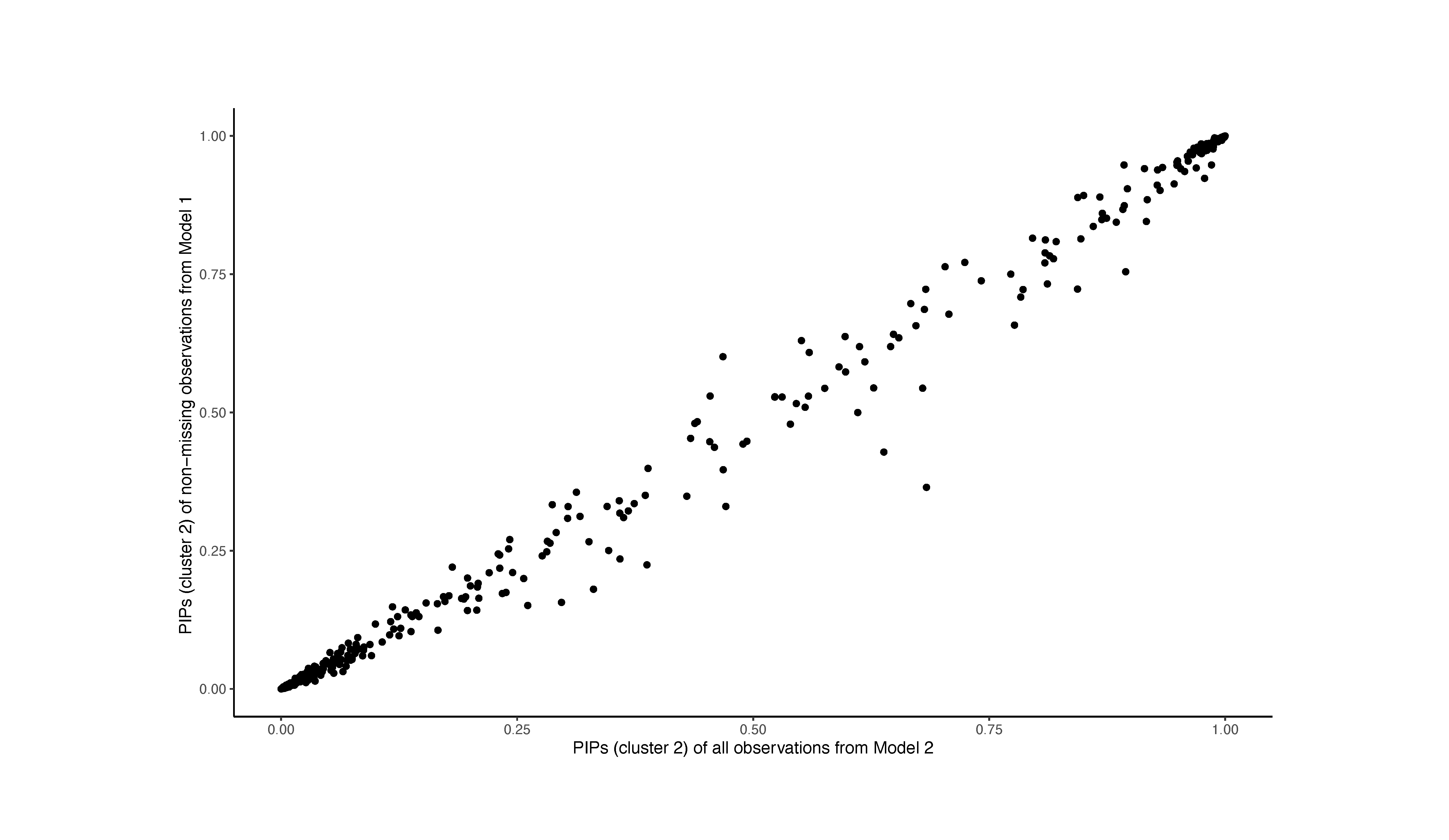

Supplement: vbae123_Supplementary_Data [file vbae123_supplementary_data.zip › Supplement Figure 2.tiff]
